# Whole cell biocatalysts: essential workers from Nature to the industry

**DOI:** 10.1111/1751-7915.12363

**Published:** 2016-05-03

**Authors:** Carla C. C. R. de Carvalho

**Affiliations:** ^1^ iBB‐Institute for Bioengineering and BiosciencesDepartment of BioengineeringInstituto Superior TécnicoUniversidade de LisboaAv. Rovisco PaisLisbon1049‐001Portugal

## Abstract

Microorganisms have been exposed to a myriad of substrates and environmental conditions throughout evolution resulting in countless metabolites and enzymatic activities. Although mankind have been using these properties for centuries, we have only recently learned to control their production, to develop new biocatalysts with high stability and productivity and to improve their yields under new operational conditions. However, microbial cells still provide the best known environment for enzymes, preventing conformational changes in the protein structure in non‐conventional medium and under harsh reaction conditions, while being able to efficiently regenerate necessary cofactors and to carry out cascades of reactions. Besides, a still unknown microbe is probably already producing a compound that will cure cancer, Alzeihmer's disease or kill the most resistant pathogen. In this review, the latest developments in screening desirable activities and improving production yields are discussed.

## Introduction

Whole cells allow the production of compounds through multi‐step reactions, with cofactor regeneration, with high regio‐ and stereo‐selectivity, under mild operational and environment‐friendly conditions. These biocatalysts are also able to carry out, for example, the selective hydroxylation of non‐activated carbon atoms which remains a challenge for classic chemistry. Besides, microbial products may be labelled as ‘natural compounds’ and fragrances and food additives can be recognized as Generally Recognized As Safe substances, increasing their value to increasingly health‐conscious consumers.

The global chemistry market was 3156 billion USD in 2013, and global sales grew by 24% when compared with 2012 (Cefic, 2014). It has been estimated that the global market for biotechnology products should grow 11.6% from 2012 to 2017 to reach 414.5 billion USD (Transparency Market Research, 2013). The total global market for microbes and microbial products reached 117 billion USD in 2012 and it is expected that by 2018 the microbial products market will be worth 174 billion USD, while the microbe market should approach 5.2 billion USD (BCC Research, 2013). The global microbial identification market alone is estimated at 896.5 million USD by the end of 2014 and to reach 1194 million USD by 2019 due to, for example, new process development, high prevalence of infectious diseases and food safety concerns (BT2606, 2014).

Biotechnological processes using whole cells require sterile initial conditions and prevention of biological contamination and should not present an advantage in one‐step biotransformation over enzymes. However, they are quite effective in multi‐step reactions, they provide a protective environment to enzymes (e.g. in non‐conventional media) and are significantly cheaper to produce than free enzymes which require several isolation and purification steps (Schmid *et al*., 2001; de Carvalho and da Fonseca, 2011). Besides, by using One Strain‐Many Compounds approach, which uses systematic alteration of cultivation parameters such as media composition, aeration rate and the use of enzyme inhibitors, it has been possible to isolate ca. 20 different metabolites from a single microorganism (Bode *et al*., 2002). Both ‘unselective’ strategies, that favour global changes in secondary metabolite production, and ‘selective’ strategies, where a specific biosynthetic gene cluster is manipulated to increase the yield of a compound, have been used for the discovery and characterization of cryptic secondary metabolites (Craney *et al*., 2013).

**Figure 1:**
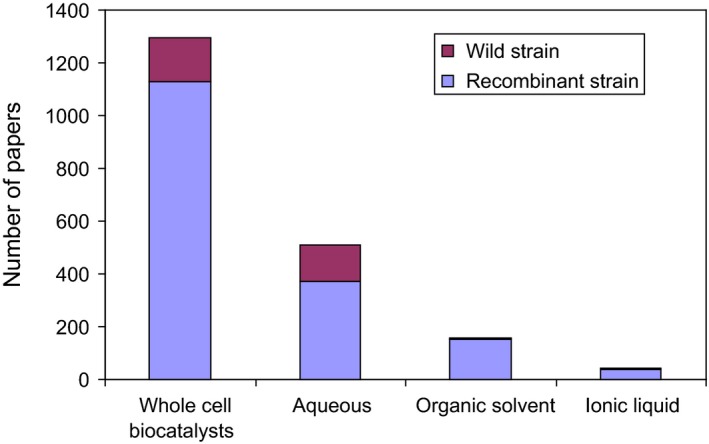
Comparison of the number of papers published on whole cells biocatalysts using wild and recombinant strains according to ISI Web of Science^™^. Number of papers using each of these types of biocatalysts in aqueous systems and in the presence of organic solvents and ionic liquids.

Genomic and metagenomic techniques have been applied to marine and terrestrial microbial samples and promised new opportunities for biodiscovery (Heidelberg *et al*., 2010), specially in environmental microorganisms that cannot yet be cultured under laboratorial conditions, through function‐based or sequence‐based screening of DNA libraries (Streit *et al*., 2004; Ferrer *et al*., 2005a; Lefevre *et al*., 2007). However, isolation of microbial cultures still provides the best approach to discover new enzymes and to develop novel processes (Mühling *et al*., 2013) and metagenomic techniques should be complemented with parallel culture libraries to fully understand the microbial diversity within a community (Donachie *et al*., 2007). Nevertheless, over 80% of the papers published on whole‐cell biocatalysts, according to ISI Web of Science^™^, report the biocatalytic activity of recombinant strains, regardless of the reaction media (Fig. 1). However, the number of industrial processes using recombinant strains is limited by problems associated with scaling up derived from genetic instability (Schwab, 1988), as well as with the ability to understand and mimic the structure, dynamics and relation between the different enzymes in complex biosynthetic pathways (Wilkinson and Micklefield, 2007).

In this review, the latest developments in screening methods to identify commercially interesting enzymatic activities in bacterial cells, and in techniques to improve whole‐cell biocatalysts, and their use in non‐conventional media are discussed. The mini‐review follows the traditional initial stages necessary to develop a biocatalytic process using whole cells: (i) biocatalyst screening; (ii) biocatalyst optimization; (iii) medium and reaction condition optimization.

## Nature's task force: screening biocatalysts from the environment

Bacteria have evolved in a myriad of environmental conditions, requiring the production of compounds, allowing their survival under such conditions and the development of metabolic routes to use the available substrates. One example is the marine environment where bacteria are often under extreme conditions of pressure, temperature, salinity and nutrient concentration, their survival being dependent on the production of biologically active compounds such as biosurfactants, siderophores, specialized fatty acids and terpenoids (de Carvalho and Fernandes, 2010). Nature, although still largely unexplored, is the most successful source of new compounds, with chemical diversity unmatched by synthetic chemistry (Watts *et al*., 2005). This is particularly true in Earth's most extreme environments.

Psychrophiles, halophiles, acidophiles, thermophiles and other extremophiles are a putative source of new enzymes and metabolites. Several biotechnology‐based companies are already taking advantage of such a pool of microorganisms, including Verenium (now part of BASF), Swissaustral Biotech SA, ZyGEM NZ Ltd and bitop AG. After isolation of bacteria, desired enzymatic activities should be tested during the discovery phase under industry‐like conditions. One of the benefits of using whole cells of halophilic bacteria, for example, is related to the fact that high salt concentrations allow non‐sterile conditions and thus low‐cost processes. Once interesting enzymes and/or metabolites are found, process development should be performed to allow the assessment of the conditions leading to high rates, yields and titres. Proper scale‐up and optimization should lead to a viable industrial process.

In recent years, metagenomic strategies have searched for biocatalysts in environmental samples without the need to isolate and cultivate individual strains. Enzymes may be discovered using: sequence‐based metagenomic approaches that look for enzymes homologous to known biocatalysts; PCR‐based methods that use primers designed according to conserved regions of known enzymes; or by functional metagenomics, where metagenomic libraries are built and screened using DNA cloned directly from environmental metagenomes (Lorenz and Eck, 2005; López‐López *et al*., 2014; Lam *et al*., 2015). The metagenomic approach has allowed the discovery of, for example, lipases and esterases (Ferrer *et al*., 2005b; Reyes‐Duarte *et al*., 2012; López‐López *et al*., 2014), amidases and amylases (Lorenz and Eck, 2005; Bashir *et al*., 2014), as well as secondary metabolites (Nováková and Farkašovský, 2013).

Functional metagenomics involves the construction of a metagenomic library, such as cosmid‐ or fosmid‐based libraries containing 25–40 kb DNA inserts, in a laborious, time‐consuming and expensive process. Although several studies have successfully identified enzymes with industrial application (Ferrer *et al*., 2005a; Lorenz and Eck, 2005; Fernández‐Arrojo *et al*., 2010; López‐López *et al*., 2014), metagenomics presents some limitations related to suitable screening strategies to find the properties sought in the target enzyme, the host organism expression and vector performance in certain hosts, which might be tackled by synthetic biology methods (Guazzaroni *et al*., 2015). Novel enzymes may be difficult to predict and detect by sequencing if no significant homology with known biocatalysts is observed, and it has been estimated that in the last decade, on average, the function of ca. 30–40% of genes in newly sequenced genomes remained unknown (Galperin and Koonin, 2010). Besides, one of the main concerns is the expression of the recovered genes in a suitable, cultivable heterologous host. Most of the studies published used *Escherichia coli* as host, but due to the low hit rates for the required targets observed in some enzyme classes, alternative hosts such as *Bacillus subtilis*,* Streptomyces* spp., *Pseudomonas* spp. or eukaryotic expression systems have also been tested (Lorenz and Eck, 2005). The large number of clones produced requires high or ultrahigh, expensive, robotic and/or liquid‐handling systems to assess those with the desired activities. Alternatively, microfluidic droplets with pico‐ to femtolitre, allowing 10^4^–10^8^ biochemical reactions per day, have been used in the discovery of novel and promiscuous enzymes at low cost (Colin *et al*., 2015; Hosokawa *et al*., 2015).

There is no question metagenomics is a powerful tool to discover novel enzymes, but several technical issues have to be tackled to convert this enormous potential into commercial success and industrial applications. Once activity has been successfully identified, the process may still be hampered if the expression of pure protein does not provide sufficient amounts of enzyme at reasonable costs.

In a different approach, several groups have tried to cultivate microorganisms that usually do not grow under laboratorial conditions. Strategies involving the reproduction of the original environment in the laboratory, growth in the presence of other bacteria and microcultivation technology in microdroplets to increase throughput, have allowed the growth of previously unculturable microorganisms (Zengler *et al*., 2002; Nichols *et al*., 2010; Stewart, 2012). In fact, the myth that fastidious and recalcitrant organisms are ‘nonculturable’ result mainly from the unwillingness of researchers to develop new media formulation, lack of patience or knowledge of past studies where intensive work solved nutritional idiosyncrasies of numerous strains (Gest, 2008, posting date; Zengler, 2009; Prakash *et al*., 2012). It is important to notice that the original microorganisms may not only contain enzymes with desirable activities but also other particularities such as adapted cellular membranes and substrate/product transport systems that may help the biocatalytic process, as discussed in the following sections. Therefore, cultivation‐based screening techniques are still relevant in a metagenomics era.

When Mühling and co‐workers tested 374 isolates of marine bacteria to assess if specific phylogenetic groups of bacteria had a higher probability of presenting particular sets of relevant enzymatic activities and to compare the distribution of enzymatic capabilities in marine and terrestrial bacteria, the authors found a larger number of novel enzyme functions than anticipated on the basis of knowledge from terrestrial bacteria (Mühling *et al*., 2013). Besides, they did not find a significant correlation between taxonomy and enzyme function, but found evidence of co‐occurrence of some enzymatic activity in the same isolate. Curiously, two enzymes that were not expected to be general enzymes, peroxidase and laccase, were very widely distributed in the 374 isolates.

Lipases are important in industry since they can carry out several reactions, including esterification, transesterification and hydrolysis. Whole‐cell lipases and esterases have received increasing importance in recent years because the organic solvent‐tolerant cells allow the production of biodiesel, for example. The immobilization of fungal mycelium on biomass support particles, and the expression of lipases on the surface of microbial cells, present as main advantage a substantial reduction in costs when compared with the use of isolated, free or immobilized enzymes (Fukuda *et al*., 2008; Xiao *et al*., 2009). The search for thermo‐ and solvent‐tolerant biocatalysts continues for the industry. A key characteristic should be their resistance to solvents such as methanol, acetonitrile, *tert*‐butyl methyl ether and toluene.

Regardless of the use of the lipases as free enzymes or inside whole cells, screening for bacterial producers is thus an important aspect in the search for novel and valuable industrial biocatalysts. For example, bacteria from the genus *Acinetobacter*,* Alcaligenes*,* Arthrobacter*,* Bacillus*,* Burkholderia*,* Pseudomonas* and *Staphylococcus* produce active lipases (Jaeger *et al*., 1994). Qualitative screening of lipase‐producing strains may be done in tributyrin (tributyrylglycerol) agar plates, with a clear zone indicating tributyrin hydrolysis (Mourey and Kilbertus, 1976). To improve the detection of lipase activity in low producers, agar plates with Tweens (fatty acid esters of polyoxyethylene sorbitan) such as Tween 80, 65, 60 or 20 as substrates, and rhodamine, Nile Blue or Victoria Blue as indicators have been proposed (Samad *et al*., 1989; Neelambari *et al*., 2011). The hydrolysis of emulsified triacylglycerols such as tributyrin, triolein and olive oil is generally applied to assess lipase activity, while soluble short‐chain fatty acid esters are usually applied to study esterase activity.

An agar plate screening method with rhodamine B‐olive oil, allowed the identification of seven thermophilic bacteria producing extracellular lipases from a Malaysian hot spring (Sheikh Abdul Hamid *et al*., 2003). The bacteria belong to the genus *Bacillus* and *Ralstonia* and the highest lipolytic activity was 4.58 U ml^−1^.

Liu and co‐authors isolated lipase‐producing strains from domestic food wastes using four media compositions, the best strategy being the use of olive oil as a substrate and COGP medium (1% olive oil, 0.4% tryptic soy broth and 1.35% agar; pH 6.0) as a selection medium (Liu *et al*., 2007). Lipase activity was determined by a pH‐stat assay, where its limitation at pH values below 7.0 was overcome by combining back‐titration and microwave treatment. This allowed the isolation of *Aeromonas* sp. C14 which presents optimal lipase activity at pH 6.0.

Angelini *et al*. (2015) developed a high‐throughput screening assay to distinguish microorganisms containing nitrilases and those possessing nitrile hydratases. A Banerjee‐modified colorimetric and pH‐sensitive assay was used with an amidase inhibitor in microplates using mandelonitrile as a substrate. The amidase inhibitor, diethyl phosphoramidate, allowed the accumulation of the amide intermediate, thus allowing the discrimination between nitrile hydratase‐amidases and nitrilases when one of these enzymatic systems was present but not when a single microorganism had both enzymatic systems. Nitrile hydratases are already used at industrial scale for the production of commodities and pharmaceuticals such as acrylamide and nictotinamide (Kobayashi *et al*., 1992; Nagasawa *et al*., 1993). *Rhodococcus* sp. N‐774, found in 1980, was the first strain to be used commercially for the production of acrylamide, but *R. rhodochrous* J1 was found to have a much more active nitrile hydratase (Nagasawa *et al*., 1993). This strain is used to produce 30 000 tonnes per year of acrylamide by Mitsubishi Rayon (Mitsubishi Rayon, pers. comm.) and to produce 6000 tonnes per year of nicotinamide by Lonza Guangzhou Fine Chemicals (Table 1). The biocatalytic industrial production of acrylamide produces less than a fifth of the CO_2_ emissions of a copper catalyst process and nearly no by‐products, and no concentration or purification steps are required, thus reducing running costs with low environmental impact (Mitsubishi Rayon, pers. comm.). Nicotinamide is produced from cyanopyridine by the immobilized whole cells of strain J1 with counter current feed under both low temperature and pressure (Meyer and Ruesing, 2008).

**Table 1:** Industrial production of pharmaceuticals and fine chemicals by whole‐cell biocatalysts

Compound	Microorganism	Production (tonnes/year)	Company	Reference
Acrylamide	*R. rhodochrous* J1	30 000	Mitsubishi Rayon	Mitsubishi Rayon, pers. comm.
Nicotinamide	*R. rhodochrous* J1	6 000	Lonza (Guangzhou)	(Meyer and Ruesing, 2008)
L‐Carnitine	*Agrobacterium/Rhizobium* *HK1349*	180	Lonza	(Meyer and Ruesing, 2008)
Violacein	*Iodobacter* sp.		Swissaustral	(Swissaustral, 2015)
Vitamin B12	*P. denitrificans* *Propionibacterium shermanii*	35	Sanofi‐Aventis, Hebei Huarong Pharmaceutical, NCPC Victor and Hebei Yuxing Bio‐Engineering	(Lee, 2014)
L‐lysine	*Corynebacterium glutamicum*	1.9 million	Ajinomoto	(Burkovski, 2015)
L‐glutamate	*Corynebacterium glutamicum*	2.9 million	Ajinomoto	(Burkovski, 2015)

A 96‐well plate system for the screening of Baeyer–Villiger monooxygenases was developed by Dudek and co‐workers, allowing the screening of large libraries of *E. coli* mutants (Dudek *et al*., 2013). For coenzyme regeneration the authors used phosphite dehydrogenase which forms phosphate during NADPH recycling, thus allowing the use of a chromogenic molybdate‐based phosphate determination assay. False positives were identified by simultaneously using a detection method for NADPH oxidases.

A microbiological method for the selection of wild‐type strains producing L‐arabinose isomerase was developed based on both the ability of the cells to produce acids and their capacity to grow on L‐arabinose (Manzo *et al*., 2013). By using 0.5% of L‐arabinose as the main carbon source and selected media, three strains could be found to be able to ferment L‐arabinose: *Enterococcus faecium* DBFIQ ID: E36, *E. faecium* DBFIQ ID: ETW4 and *Pediococcus acidilactici* ATCC ID: 8042. L‐arabinose isomerase activity in cell‐free extracts and in saline‐precipitated cell‐free extracts of these strains, determined by the cysteine carbazole sulphuric acid method, indicated *E. faecium* DBFIQ ID: E36 as the best biocatalyst.

## Improving microorganisms

Although Nature is still the best source for novel biocatalysts, most do not present sufficient abilities in terms of productivity, stability and availability to be used in industrial processes. Besides, some molecule or environmental signal might be necessary to trigger the production of genetically encoded compounds that are not produced under laboratorial conditions: for example, the complete genome sequence of actinomycetes indicates that ca. 90% of the putative metabolites are still undiscovered (Wilkinson and Micklefield, 2007). New techniques such as recombinant DNA, metabolic engineering and combinatorial biosynthesis allow the improvement of industrial processes while genomics, genome mining, proteomics and metabolomics have led to the discovery of novel products (Table 2). Metabolic fluxes and their in vivo control aim at pathway design, construction and optimization of biocatalysts for the cost‐effective production of fuels and fine chemicals (Woolston *et al*., 2013; Yadav and Stephanopoulos, 2014). In inverse metabolic engineering, which started to be used in the early 2000s, combinatorial metabolic engineering is used for the isolation of specific mutations that result in the improved phenotype of the whole cell. The success of this technique is thus dependent on the level and quality of the genetic diversity that may be generated (Skretas and Kolisis, 2012).

**Table 2:** Examples of genetic techniques used in biocatalysis

Technique	Rational	Examples
Genome mining	DNA sequences encoding for enzymes involved in the synthesis of desired products are searched in the genome	Ferrer *et al*. (2005a), Wilkinson and Micklefield (2007)
Metagenomics	Study of genetic material recovered directly from environmental samples	Lorenz and Eck (2005), Fernández‐Arrojo *et al*. (2010)
Directed evolution	After DNA mutagenesis, the fittest variants with a desired phenotype are selected from a series of mutants	Grosse *et al*. (2010), Derkx *et al*. (2014)
Recombinant DNA	The DNA sequence encoding for an enzyme is cloned into an expression vector and transferred into a production host	Matsuyama *et al*. (2002), Kayser (2009), Lee *et al*. (2015)
Metabolic engineering	Production of a compound is improved by optimizing genetic and regulatory processes in the cell	Stephanopoulos (1999), Papagianni (2012), Fisher *et al*. (2014)
Combinatorial biosynthesis	Manipulation of enzymes and pathways to create new ‘unnatural’ products or natural product analogues	Shibamoto *et al*. (2004), Skretas and Kolisis (2012)
Biosynthetic engineering/ synthetic biology	Design and fabrication of biological components and systems that may or may not exist in the natural world	Wilkinson and Micklefield (2007), Winter and Tang (2012), Turconi *et al*. (2014)

In recent years, extremophiles, including thermo‐, acido‐, alkalo‐, psychro‐ and piezophiles, have become an important source of enzymes (Gupta and Khare, 2009; Oren, 2010; Liszka *et al*., 2012; Elleuche *et al*., 2015; Leal Dalmaso *et al*., 2015). Since wild‐type extremophile strains rarely produce enzymes in significant yields, it became standard to clone the genes encoding the desired enzymes in well‐established expression hosts, such as *E. coli*,* P. pastoris* and *B. subtilis*. Metagenomic approaches even allow the screening of enzymes from environmental gene pools by cloning their sequences into suitable hosts without the necessity to cultivate the original strains (Ferrer *et al*., 2005a; Moe *et al*., 2010).

Using genome mining, cloning and directed evolution, Grosse and co‐workers compared the thermostability of two highly enantioselective esterases from *Bacillus cereus* and *Thermoanaerobacter tengcongensis* derived from the natural biocatalyst and from genetic variants made in the laboratory (Grosse *et al*., 2010). The *S‐*specific esterase from *B. cereus* is able to perform the racemic resolution of O‐benzyl lactic acid ethyl ester, which is a key chemical intermediate in the synthesis of the potent antibiotic, levofloxacin. The enzyme was identified, cloned and expressed in *E. coli*, and the authors used directed evolution to improve the thermostability of the biocatalyst. Similarly, a predicted open reading frame with a presumed hydrolase or acyltransferase function from thermophile *T. tengcongensis* was also cloned and characterized. Although two improved variants were produced with a 3–5°C increase in the apparent melting temperature over the native esterase (T_m_ of 50°C), they were outperformed by the naturally alkaliphilic esterase homologue (T_m_ of 65°C). As mentioned by the authors, the study provides a rare example of a naturally occurring thermostable biocatalyst that performs better than the homologues produced by directed evolution.

Metabolic engineering by means of recombinant DNA technology result in a large number of genetic variants to be tested. According to Meyer *et al*., most laboratories working on metabolic engineering at the millilitre scale can easily generate libraries with up to 19^9^ genetically different isolates (Meyer *et al*., 2015). Since in industrial settings, high‐throughput screening assays include up to 10^4^ variants per evolution round, the authors proposed a method based on microcompartmentalization. Cells of *B. subtilis* were tested as biocatalysts of the biotransformation of cellobiose into vitamin B2 in gel capsules that worked as nanolitre reactors. *E. coli* cells present in these reactors were used as sensor cells since they produce GFP as a concentration‐dependent response to B2. GFP fluorescence intensity could thus be used to indicate the most efficient *B. subtilis* variants to produce B2 from cellobiose.

The genes of two enzymes, DmpA from *Ochrobactrum anthropi* and 3‐2W4 BapA from *Sphingosinicella xenopeptidilytica*, which may be applied to the synthesis of *β*‐ and *β*,*α*‐peptides such as L‐carnosine, were expressed in *E. coli* and *P. pastoris* (Heyland *et al*., 2010). The recombinant strain, *E. coli* DmpA_syn_, could be used for the synthesis of L‐carnosine directly as a whole‐cell biocatalyst, thus reducing the time‐consuming and material‐intensive protein purification process. Besides upstream simplification, downstream processing could also be simplified, thus decreasing environmental and economical costs. By optimization of the reaction conditions, including pH and substrate concentration, the performance of the recombinant *E. coli* strain could be improved to allow yields of L‐carnosine up to 71%. In a fed‐batch process, with at least five repeated batches, the recombinant *E. coli* strain allowed the accumulation of 3.7 g L^−1^ of L‐carnosine.

Sjostrom *et al*. (2014) developed a high‐throughput method to screen a yeast cell library with 10^5^ members with diverse phenotypes to assess the best α‐amylase production. The method involved the encapsulation of single cells with a fluorogenic reporter substrate in 20 μl microfluidic droplets, allowing over 300 times throughput and a million‐fold decrease in reagent consumption than automated microtitre plate‐screening systems. This allowed the discovery of a clone with more than two times the α‐amylase production of the original strain.

Another strategy used may involve the inhibition of metabolic pathways to increase the production of the desired compound by a modified strain. Yu *et al*. (2014) genetically constructed the strain *P. putida* P‐HSP to accumulate 6‐hydroxy‐3‐succinoyl‐pyridine, an intermediate in nicotine degradation, by blocking the catabolic pathway of nicotine. Homologous recombination was used to disrupt the *hspB* gene in strain, *P. putida* S16, which is necessary to convert 6‐hydroxy‐3‐succinoyl‐pyridine into 2,5‐dihydroxy‐pyridine. The modified strain produced 6.8 and 16.3 g L^−1^ of the desired compound from respectively tobacco‐waste and nicotine.

Biosynthetic engineering even allows the production of unnatural metabolites by rational manipulation of biosynthetic pathways or by the combination of more than one pathway to generate a hydrid product (Wilkinson and Micklefield, 2007; Winter and Tang, 2012). Pathway engineering using synthetic biology requires enzymes to be used in vivo and not isolated, especially if complex enzymes such as the alkane mono‐oxygenase (AlkB) of *Pseudomonas putida* GPo1, which requires two coenzymes and two cofactors, are envisaged (Grant *et al*., 2014). There is still a debate if synthetic biology is a new discipline of engineering, an extension of biotechnology or actually just another name for genetic engineering (Collins, 2014).

The design of the microbial cell factories involves several steps, including: identification of a de novo biosynthetic pathway for the desired product; selection of a microbial chassis (host); enzyme and metabolic engineering to allow the formation of the new products in the microbial cell factory host (Pscheidt and Glieder, 2008; Fisher *et al*., 2014; Zhang *et al*., 2015). The strategies for the production of the desired compound(s) in microorganisms involve: the application of heterologous genes to assemble a new biosynthetic pathway in the host; improvement of the pathway flux by, for example, augmenting substrate availability, down‐regulating competing pathways or increasing the expression of key enzymes; the improvement of certain enzymes of the pathway by protein engineering (Pearsall *et al*., 2015).

The use of hosts for biosynthesis still presents several difficulties in the most commonly used species such as: the post‐transcriptional modification and metabolic capabilities of *E. coli* are limited and product extraction may be difficult if the protein products are secreted into the periplasm or form inclusion bodies; *E. coli* may produce an endotoxin lipopolysaccharide that may cause fever in humans if not completely removed from products; *B. subtilis* is endotoxin‐free and secretes proteins into the extracellular medium, but there is an insufficient number of expression vectors and this species presents, for example, plasmid instability, misassembled proteins and active proteases (Lam *et al*., 2012). Besides, many parts of DNA sequences are usually not fully characterized and when they are, their performance may be different or may cease when placed in a distinct cell type, under laboratorial conditions or when assembled with other sequences (Kwok, 2010). Nevertheless, *E. coli*,* Saccharomyces cerevisiae*,* Corynebacterium glutamicum* and *Pseudomonas* sp. have been successfully used as microbial hosts with the current genetic tools available (Pearsall *et al*., 2015).

To test and construct large synthetic circuits, where it is necessary to find the genes involved in the pathway and to develop control systems for their correct expression, the man‐hours necessary may be enormous. To produce the most successful compound developed by synthetic biology, the antimalarial drug precursor, artemisinin, it was estimated that ca. 150 person‐years of work were necessary, which were supported by two grants totalling 53.3 million USD awarded by the Bill & Melinda Gates Foundation (Kwok, 2010; Paddon *et al*., 2013). The engineered *S. cerevisiae* produces 25 g L^−1^ of artemisinic acid which is chemically converted to artemisinin in a semi‐synthetic process that resulted in the production of 35 tonnes of artemisinin in 2013 and 60 tonnes in 2014 by the company Sanofi (Turconi *et al*., 2014). Artemisinin was first isolated from the plant *Artemisia annua* and tested as antimalarial drug by Tu Youyou who was awarded the Nobel Prize in Physiology or Medicine in 2015. The production of artemisinin by a semi‐synthetic process has opened a debate regarding its impact on *A. annua* farmers (Thomas, 2013).

Cell surface display permits the addition of proteins, peptides or other molecules to the surface of microbial cells, which may be used to improve biocatalytic activities (Smith *et al*., 2015). Recently, a xylanase from *Thermomyces lanuginosus* DSM 5826 was fused to the surface of *E. coli* resulting in the ability of the latter to degrade xylan (Qu *et al*., 2015). An activity of ca. 70 U/g dry cell weight was achieved by the *E. coli* cells at pH 6.2 and 65°C. Shibamoto *et al*. (2004) created a combinatorial yeast library through cell surface display of the pro‐ and mature region of lipase from the fungus *Rhizopus oryzae*. This technique allowed a rapid screening of active engineered enzymes, and the clones produced could be applied as whole‐cell biocatalysts in industrial processes.

In industries, such as the food industry with increasingly health‐driven consumers and strict legislation, the use of recombinant DNA technology to improve microbial performance is not an alternative, and classical methods may be used. Lactic acid bacteria, used for the production of, for example, yogurt and cheese, have been improved by random mutagenesis, directed evolution and dominant selection (Derkx *et al*., 2014). Although in the latter two cases mutagens are not usually required, multiple mutations may still occur and the new metabolites produced may be toxic to humans. Nevertheless, lactic acid bacteria have been receiving increased attention as cell factories for the production of compounds for the food and pharmaceutical industries, in particular *Lactococcus lactis* (Papagianni, 2012).

### Non‐genetic improvement of biocatalysts

When whole‐cell biocatalysts are used, substrate(s) have to cross the cell envelope to reach the enzyme(s) and the reaction rate may be decreased when compared with free enzymes. Several studies have shown that it is possible to improve substrate transfer across cell walls and membranes by increasing their permeabilization level by chemical (with, for example, detergents and solvents) or physical (e.g. temperature shock) methods. Since these methods may damage the cell integrity, may cause leakage of cellular components and affect downstream processes, the best fluidity level should be sought.

To improve fatty acid methyl ester production for biodiesel‐fuel production with *R. oryzae* cells, Hama and co‐workers studied the effect of the membrane fatty acid composition on the lipase transesterification activity (Hama *et al*., 2004). By adding several fatty acids to the culture medium, they could influence the degree of saturation of the cellular membrane. Oleic or linoleic acid‐enriched cells presented higher activity than those with saturated fatty acid‐enriched membranes, while palmitic acid‐enriched cells showed significantly higher enzymatic stability. Higher membrane permeability thus increased activity and higher membrane rigidity favoured enzymatic stability. The authors found that a ratio of 0.67, calculated by the ratio of oleic acid and the sum of oleic and palmitic acids, allowed both good transesterification activity and enzymatic stability.

Permeabilized cells even enable the use of externally added cofactor(s), as demonstrated by Zhang *et al*. (2006) who have coupled two permeabilized microorganisms: *B. pumilus* Phe‐C3 catalysed the reduction of ethyl 3‐keto‐4,4,4‐trifluorobutyrate, while *B. subtilis* BGSC 1A1 did the cofactor regeneration by converting glucose to gluconolactone. The cells, permeabilized by exposure to 5–7% toluene and 5 mM EDTA, could yield 89% of the (*R*)‐hydroxyester and be reused. Besides, NADPH could be recycled more than 1600 times.

Adaptation of the fatty acid composition of the phospholipids of the cellular membrane of *R. erythropolis* cells, with concomitant alterations in the net surface charge and cell hydrophobicity, in the presence of toxic substrates, products and solvents used in organic:aqueous systems, also resulted in increased biotransformation rates and yields (de Carvalho *et al*., 2005a; de Carvalho *et al*., 2007, 2009, 2014a). These cells could also be rapidly adapted to high salt concentrations, being able to change the fatty acid composition in the 30 min following exposure (de Carvalho *et al*., 2014a). However, the most remarkable feature was that this species could be adapted to grow and to metabolize C6‐C16 *n*‐alkanes and alcohols under conditions that were regarded as extreme conditions for this bacterium (de Carvalho, 2012). Non‐adapted cells could not grow at temperatures below 15°C or above 35°C, at pH values below 4 or above 9 and at concentrations higher than 5.5% (w/v) sodium chloride or 0.4% (w/v) copper sulphate. A stepwise adaptation strategy allowed the growth of these cells at 4–37°C, pH 3–11, and in the presence of up to 7.5% salt and 1% copper sulphate. The cells changed the relative proportion of straight, methyl and cyclopropyl saturated, unsaturated and hydroxyl‐substituted fatty acids and produced polyunsaturated fatty acids unusual in bacteria. This study thus demonstrated that it is possible to take a bacterium that is able to convert a wide array of substrates, and to adapt it to grow and convert substrates under conditions far from optimal.

## Using whole cells in non‐conventional media

A large number of potentially interesting substrates and products for industrial production are lipophilic, presenting low water solubility. One way to overcome this drawback is the use of organic solvents which act as substrate and/or product reservoir. Although the action of porcine pancreatic lipase was described in the 1930s by the Polish scientist Ernest A. Sym (Sym, 1930, 1936), the dogma that enzymes are active only in aqueous media prevailed over the next 40–50 years (Halling and Kvittingen, 1999; Klibanov, 2000). Water is necessary for enzyme activity due to its role in the maintenance of the native catalytically active conformation of the enzyme, being involved in the formation of hydrogen bonds and in van der Waals interactions. But the amount of water necessary may be as little as a monolayer of water molecules surrounding the enzyme (Zaks and Klibanov, 1985, 1988). In fact, the vast majority of the papers being published report the use of enzymes and whole cells in organic media and ionic liquids (de Carvalho, 2011). In the presence of organic solvents, remarkable changes in region and enantioselectivity of the enzymes are often observed, and synthetic reactions are favoured. To further favour the reaction equilibrium towards synthesis, aqueous‐organic, organic, and gas–solid systems have been tested and implemented (Lamare *et al*., 2004; de Carvalho and da Fonseca, 2011; Stepankova *et al*., 2014).

Solvent‐tolerant microorganisms provide an efficient solution to biocatalytic systems involving nearly water insoluble compounds (León *et al*., 1998; Sardessai and Bhosle, 2004; Tang *et al*., 2009; Torres *et al*., 2011). By providing a natural environment to enzymes, whole cells prevent the loss of activity through conformational changes in the protein structure that are often observed in non‐conventional medium. Some bacterial species, including those from the genus *Rhodococcus* and *Mycobacterium*, can even present higher activity and viability in the presence of organic solvents than in aqueous systems (de Carvalho and da Fonseca, 2002; de Carvalho *et al*., 2004). Besides the ability to survive in the reaction media, an efficient whole‐cell biocatalyst must allow the transport of both substrate(s) and product(s) across the membrane, should not consume either of them in side reactions and should be able to recycle the necessary cofactors.

In the case of toxic substrates, they may be fed to the bioreactors at limiting rates, so that they are transformed prior to their accumulation and non‐toxic concentrations are achieved, while toxic products may be removed soon after they are produced. In situ product removal (ISPR) may be achieved by using auxiliary phases, for example, extractants or adsorbents, but which may have difficulties in accumulating products with intermediate polarity to commercially interesting amounts (Straathof, 2003). Similarly, substrate toxicity may be prevented by in situ substrate addition (ISSA), with the substrate concentration being controlled by mass transfer from the auxiliary to the aqueous phase (Straathof, 2003).

Using both ISPR and ISSA, it was possible to produce limonene‐1,2‐diol and simultaneously carry out the diastereometric resolution of limonene‐1,2‐epoxide with *R. erythropolis* DCL14 cells (de Carvalho *et al*., 2000). A 500 ml fed‐batch mechanically stirred reactor was used with an external loop for the recirculation of the aqueous phase through a LiChroprep RP‐18 containing column. Since the cells of strain DCL14 are very hydrophobic, they attached to the organic phase allowing the recirculation of the aqueous phase which was almost cell free. At the end of the experiment, *trans*‐epoxide could be recovered from the organic phase by vacuum distillation of the solvent. The diol and *trans*‐epoxide adsorbed on the RP‐18 column could be recovered by elution with 40% and 100% acetone respectively.

Two solvent‐tolerant *Bacillus* strains, SB1 and BC1, were able to convert cholesterol to cholest‐4‐ene‐3,6‐dione in a 1:1 chloroform:phosphate buffer system (Sardessai and Bhosle, 2003). The *Bacillus* strains were isolated from Arabian Sea sediment and besides being able to convert cholesterol, they also presented excellent solvent tolerance in particular to chloroform.

Steroid‐based pharmaceuticals are used extensively as, for example, antitumour, anti‐inflammatory, antimicrobial and antiallergy compounds, anabolic and contraceptive agents, and for the prevention and therapy of several impairing diseases such as hormone‐dependent forms of breast and prostate‐cancer, rheumatoid arthritis, hypertension, obesity, diabetes, neurodegenerative diseases and metabolic disorders (Fernandes *et al*., 2003; Donova and Egorova, 2012). Actinobacteria are particularly efficient in biocatalysis of steroids, being able to carry out dehydrogenation, oxidation of steroid alcohols, double bond isomerization and hydrogenation, reduction in steroid ketones, deacetylation, hydroxylation and partial or complete degradation of the side‐chain of steroids (Donova, 2007). Besides, bacteria from this phylum, which include the genus *Streptomyces*,* Mycobacterium* and *Rhodococcus*, are particularly apt in non‐conventional media. Free resting cells of *Mycobacterium* sp. NRRL B‐3805 presented higher cell viability and activity in the side‐chain cleavage of β‐sitosterol in biphasic systems containing bis(2‐ethylhexyl) phthalate (BEHP) than in systems containing only phosphate buffer (de Carvalho *et al*., 2004). Curiously, when the solvent droplets were observed under the microscope, the cells were found adhered to the surface of the solvent droplets, but no cells could be observed inside them. Nevertheless, in pure BEHP, 95% of the cells were able to remain viable for at least 150 h and increased by 19% and 14% the activity when they were pre‐incubated for 6 and 12 h, respectively, with the solvent prior to the addition of the substrate.

The cleavage of the side‐chain of β‐sitosterol is a well‐established, industrial, multi‐enzymatic process involving the use of nine catabolic enzymes in a 14‐step metabolic pathway (Fernandes *et al*., 2003), and therefore, green solvents should be preferably used. Marques *et al*. (2010) compared the biocatalytic performance of strain NRRL B‐3805 in ionic liquids, polyethylene glycol (PEG), polypropylene glycol (PPG), UCON^™^ (commercial combination of PEG/PPG) and silicone to that obtained in systems containing dioctyl phthalate. *Mycobacterium* sp. cells converted all β‐sitosterol in 120 h when silicone was used. Cells in phthalate and PPG400 converted 3.17 and 2.89 mM of the initial 12 mM substrate, respectively, while cells in the system with the ionic liquid EMIM‐EtSO4 converted only 2.32% after 5 days. Besides, the cells were able to maintain the maximum reaction rate during a 50‐fold scale‐up of the bioconversion system with silicone, based on the maintenance of the power consumption between scales.

The first bioconversion requiring cofactor regeneration performed in the presence of ionic liquids, acting as a substrate reservoir and product extraction phase, was the asymmetric reduction of 4‐chloroacetophenone to (*R*)‐1‐(4‐chlorophenyl)ethanol by *Lactobacillus kefir* (Pfruender *et al*., 2004). Although the possibility of fine‐tuning the physicochemical properties of ionic liquids makes them desirable solvents, their composition at relatively low temperatures and toxicity towards microbial cells and superior organisms are challenging the ‘green label’ that they used to carry (Quijano *et al*., 2010). Nevertheless, *S. cerevisiae* is known to synthesize industrially relevant alcohols and ketones (Quijano *et al*., 2010) and *Rhodococcus* sp. to biotransform nitriles (Cull *et al*., 2000) in the presence of these solvents.

## Resting versus growing cells

The use of resting cells may be a good alternative when the best pH value, temperature or media composition for the bioconversion is different from the values allowing the best growth conditions. In this situation, the cells are grown until enough biomass has been accumulated, they are harvested and washed with water or a buffered solution and resuspended in the desired buffer for biocatalysis. Since the cells are washed, unconsumed growth substrates and nutrients, as well as undesired growth metabolites are removed from the system allowing better product recoveries and downstream processing. Besides, resting cells can show high product yields on carbon and energy sources since they are not used for biomass production, and the cells may be recycled and reused. However, these cells should be able to maintain high activities and cofactor regeneration over extended periods of time to be used in biocatalytic systems.

Julsing *et al*. (2012) reported the epoxidation of styrene by resting cells of *E. coli* containing the styrene monooxygenase genes *styAB* from *Pseudomonas* sp. VLB120, with the cells doubling the specific activity and presenting high glucose yields when compared with growing cells. Kiss *et al*. (2015) could also efficiently scale‐up the conversion of cyproterone acetate to its main human metabolite, 15β‐hydroxycyproterone acetate, by *Bacillus megaterium* ATCC 13368 which contains the enzyme CYP106A2. Product formation reached 0.43 g L^−1^ and no significant differences could be observed between growing and resting cells.

Resting whole cells of *Rhodococcus* strains have also been found able to efficiently carry out several biotransformations, including: hydrolysis of nitriles and acid amides such as the hydrolysis of racemic naproxen amide (*RS*)‐1 to enantiomerically pure naproxen (*S*)‐2; desulfurization of dibenzothiophene to 2‐hydroxybiphenyl; and, 9 alpha‐hydroxylation of 4‐androstene‐3,17‐dione (de Carvalho and da Fonseca, 2005; de Carvalho, 2011). However, during the adaptation of *R. erythropolis* DCL14 cells to solvent, substrate and product, to improve the biocatalytic production of carvone from carveol, it was found that the cells presented higher maximum carvone production rates in mineral medium than in phosphate buffer, indicating that a regenerative medium is necessary during the adaptation period and thus growth of the most apt individuals should be required (de Carvalho *et al*., 2005b). Curiously, high carvone production periods were followed by periods during which almost no carvone accumulation occurred, and these were followed by other periods of high carvone production. The duration of productive and non‐productive periods was similar for the same initial incubation period.

The cells also react differently to reaction conditions. By choosing the operating conditions, Cantarella *et al*. (2006) were able to selectively control the nitrile hydratase‐amidase system in the resting cells of *Microbacterium imperiale* CBS 498‐74. When a batch reactor was used, benzonitrile was converted to benzamide and benzoic acid, and the accumulation of benzoic acid demonstrated that the reaction could not be stopped at the end of the first reaction step. By using a UF‐membrane bioreactor at 20°C and a residence time of 10.3 h, the cells converted 96.9% of benzonitrile into benzoic acid, but at 5°C and with a residence time of 22.5 h, the cells converted 70.5% of the substrate into benzamide. The different temperature dependence of nitrile hydratase and amidase in the resting cells and an efficient choice of residence time thus allowed the control of the reaction system.

## Concluding remarks

High‐throughput screening and microbiological techniques aimed at improving biocatalyst performance are increasing the interest of biocatalysis using whole cells by the industry. There is a huge potential for the finding of novel enzymatic activities in marine and extreme environments as technologies improve to access such locations. Fast screening, strain improvement and integrated bioprocess design are thus expected to provide high‐value products using environment‐friendly processes in the near future.
